# Evidence of different predictors for different types of cardiotoxicities after hypo-fractionated whole breast radiotherapy

**DOI:** 10.3389/fonc.2026.1893940

**Published:** 2026-07-22

**Authors:** Alfonso Belardo, Andrei Fodor, Marco Fois, Kerby Bjorn Dimayuga, Lucia Perna, Laura Giannini, Paola Mangili, Gabriele Palazzo, Marcella Pasetti, Miriam Torrisi, Roberta Tummineri, Antonella Del Vecchio, Tiziana Rancati, Nadia Gisella Di Muzio, Claudio Fiorino

**Affiliations:** 1 Medical Physics, IRCCS San Raffaele Scientific Institute, Milan, Italy; 2 Radiation Oncology, IRCCS San Raffaele Scientific Institute, Milan, Italy; 3 Data Science Unit, Fondazione IRCCS Istituto Nazionale dei Tumori, Milan, Italy; 4 Faculty of Medicine and Surgery, Vita-Salute San Raffaele University, Milan, Italy

**Keywords:** breast, cardiac calcification, cardiotoxicity, events correlation, radiotherapy

## Abstract

**Background and purpose:**

Cardiac calcifications (CAC) are emerging as relevant predictors of cardiac toxicity after breast cancer radiotherapy. This study aimed to investigate the association between quantitative CAC scores and specific subgroups of cardiac events in patients treated with moderate hypofractionation, and to assess the interplay between dosimetric and clinical predictors for different families of events.

**Materials and methods:**

Data from 1172 consecutive breast cancer patients treated at a single institution with 40 Gy in 15 fractions were analyzed. Mean heart dose (MHD) was extracted after automatic heart segmentation. Agatston score (AS), CAC volume, and maximum heart HU value (Max_HU) were quantified from planning CT scans. Available clinical variables included laterality, chemotherapy, age, smoking status, and respiratory toxicity occurring within 3 years after radiotherapy. Cardiac events were classified into two groups: CAC_corr (coronary artery disease, acute myocardial infarction, congestive heart failure, and valvular disease) and CAC_uncorr (conduction abnormalities, atrial premature beats, atrial fibrillation, pericarditis, and tachyarrhythmia). Associations between predictors and outcomes were tested using logistic regression.

**Results:**

After excluding patients with pacemakers/electrodes, 1091 patients were available. With a median follow-up of 6.5 years, 29 cardiac events were recorded. Among them, 15/29 were CAC_corr and were similarly distributed between right-and left-sided tumors (8 vs 7). Conversely, 13/14 CAC_uncorr events occurred in left breast cancer patients; therefore, analyses for this subgroup were restricted to left-sided cases (13/561). For CAC_corr events, the combination of AS/CAC_volume/Max_HU with age showed the best predictive performance (AUC=0.80/0.79, p<0.0001), while respiratory toxicity had no impact. For CAC_uncorr events, age and anthracycline-based chemotherapy (FEC) were the strongest predictors (AUC=0.73, p=0.0125). Including respiratory toxicity significantly improved model performance (AUC=0.77, p=0.0003). MHD was not independently associated with cardiac events.

**Conclusion:**

CAC_corr events were mainly associated with CAC burden and age, independently of tumor laterality. In contrast, CAC_uncorr events appeared more related to treatment-related factors, as most occurred after left-sided irradiation. Within left breast cancer patients, these events were mainly associated with age, anthracycline-based chemotherapy, and respiratory toxicity rather than with MHD.

## Introduction

Breast cancer remains the leading cause of cancer-related mortality among women in Europe (1). However, mortality rates have steadily declined over recent decades, largely due to improvements in early diagnosis and therapeutic strategies (2). Therefore, the population of long-term breast cancer survivors is continuously growing, making quality of life (QoL) an increasingly relevant issue for these patients. Radiotherapy (RT) following breast-conserving surgery represents a cornerstone of breast cancer management and is delivered to most patients (3). Advances in treatment planning and delivery techniques enabled a significantly improved sparing of surrounding organs at risk (OARs), particularly the lungs and the heart. Nevertheless, non-negligible radiation doses to OARs may be delivered, potentially leading to radiation-induced toxicities with a negative impact on patients’ QoL (4). Moreover, the unavoidable irradiation of the skin and breast tissues can result in both transient and long-term adverse effects (5–9).

In recent years, increasing attention has been devoted to long-term cardiovascular diseases (CVD (10, 11)). Notably, cardiac-related mortality has been reported to be higher in women treated for breast cancer compared to the general female population without breast cancer (12). A linear dose–response relationship between mean heart dose (MHD) and the risk of CVD has been suggested (10), pushing a growing emphasis on heart-sparing strategies over the past decade. This heightened awareness resulted in a substantial reduction of MHD in contemporary treatment series (13) and, consequently, in a much more blurred association between MHD (or other cardiac dosimetric parameters) (14–16) and CVD in modern series. On the other hand, several non-dosimetric factors are known to significantly modulate cardiovascular risk, including exposure to anthracyclines, the presence of chronic kidney disease, and smoking habits (15–18).

In addition, pre-radiotherapy cardiac and vascular symptoms are, as expected, associated with an increased risk of cardiovascular disease (17, 18). Similarly, the lung parenchyma, partially included within the tangential breast fields, can be exposed to clinically relevant radiation doses, predisposing patients to radiation-induced pneumonitis and, in some cases, to persistent fibrosis (19, 20). Any interplay between respiratory events and CVD cannot be excluded in principle and has never been investigated for breast cancer patients, at our knowledge.

More recently, cardiac calcifications (CAC) emerged as strong predictors of CVD and worse survival outcomes (21), even in patients who are clinically asymptomatic (22–26). The most widely adopted metric for stratifying cardiovascular risk according to CAC burden is the Agatston Score (AS (27, 28),), which integrates both the density and the spatial extent of calcified plaques. As is not currently suitable for the clinical setting, alternative and more easily quantified scores, such as maximum CAC density (HU score) and CAC volume, have been explored (21). Interest in CAC assessment is steadily increasing, as it offers the potential to identify asymptomatic patients at higher cardiovascular risk, thereby supporting treatment plan optimization, guiding radiation delivery strategies, and enabling the selection of patients who may benefit from dedicated monitoring programs aimed at the prevention, early detection, and timely management of cardiovascular complications (29, 30).

The potential interaction between cardiac radiation dose and the presence and severity of CAC in identifying patients at increased cardiovascular risk remains insufficiently investigated. Van der Bogaard et al. (31) were among the first to report a more pronounced association between cardiac dosimetric parameters and acute coronary events in patients without CAC in the left anterior descending artery (LAD). More recently, another large study suggested that the AS may represent a stronger predictor of cardiovascular risk than MHD, particularly in node-positive patients treated with irradiation of the whole breast, supraclavicular nodes, axilla, and with or without internal mammary node irradiation (26).

Leveraging a large institutional database of patients treated at our Institute most of whom received hypofractionated RT several investigations have been performed addressing both clinical outcomes and treatment-related toxicities (9, 21, 31–35).

The availability of complete treatment planning data and long-term follow-up provided the opportunity to move beyond a global assessment of cardiac risk and permitted the confirmation of CAC scores as major predictors of CVD in patients treated with moderate hypofractionation (40 Gy/15 fractions) to the whole breast only (21). Moving forward, in current study we aimed to investigate whether CAC burden could help differentiate events from those potentially more attributable to radiation exposure; then, the aims of the current investigation were:

- to test the association between CAC scores and specific subgroups of cardiac events, with the purpose of identifying events predominantly linked to pre-existing conditions and likely to be not (or poorly) caused by Radiotherapy.
- to assess interplay with dosimetry/clinical predictors and with the presence of respiratory toxicities.

## Materials and methods

### Patient cohort: clinical and dosimetry characteristics

Data was retrieved from a prospectively maintained institutional database including all breast cancer patients treated with postoperative radiotherapy since 2009. For the purposes of the present analysis, we selected patients who received a hypofractionated regimen (40 Gy in 15 fractions) to the whole breast only, excluding those with nodal irradiation or a boost to the surgical bed. To ensure an adequate follow-up duration and considering that the most recent update was available up to December 2023, only patients treated before 2018 were included, thus allowing for a minimum follow-up of 5 years after radiotherapy.

The initial cohort consisted of 1200 patients; of these, 28 were excluded due to bilateral radiotherapy (either synchronous or sequential), resulting in a final study population of 1172 patients (right-sided: 569; left-sided: 603). Details regarding contouring, treatment planning, and delivery have been reported elsewhere (32, 34). Briefly, all patients underwent CT simulation on the same scanner (Light Speed GE MDCT) and were treated with manually optimized three-dimensional conformal radiotherapy (3D-CRT) using tangential fields.

The clinical target volume (CTV) encompassed the whole breast, in accordance with national guidelines (36). The planning target volume (PTV) was generated by expanding the CTV by 5–10 mm in all directions, except towards the lung (5 mm), and was cropped to exclude the first 5 mm beneath the skin surface. Dose homogeneity within the PTV was achieved using a median of four segments, with planning objectives of D1% < 108% and V95% > 95%. Target delineation and treatment techniques remained consistent over the study period. Radiotherapy was delivered using a Varian Linac DHX (6 MV), with daily image guidance performed via cone-beam CT (CBCT).

The main characteristics of the patients are summarized in **Table 1**.

**Table 1:** Summary of the major clinical characteristics of the patients.

Total number of patients	1091
Age groups	
Mean Median [IQR] < 45	60 62 [50;70] 114
45 - 55	278
55 - 65	268
> 65	431
Position of tumour	
Left	561
Right	530
Chemotherapy	
Anthracycline based	180
Other	96
Concurrent Chemo/RT	27
Other therapies	
Trastuzumab	77
Hormonal	879
Health conditions	
Obesity	251
Diabetes	150
Hypertension	376
Smoking	221
Mean Heart Dose (MHD)	
Average (SD) [Gy]	1.72 (1.23)
Patients with MHD > 1 Gy	595

Smoking status was categorized based on patient-reported history: individuals were defined as active smokers if they were smoking at the time of anamnesis, regardless of the number of cigarettes, and as former smokers if they had quit prior to anamnesis, irrespective of the duration of cessation. For this analysis, active and former smokers were grouped together.

Comorbidities were also collected during anamnesis; in particular, previously diagnosed obesity, diabetes, and hypertension were self-reported by patients and included in the analysis.

Pulmonary events occurring after RT were collected following the same procedural framework adopted for cardiac toxicity surveillance described in detail in (32). Specifically, Actinic pneumonia/pulmonary fibrosis was registered at each scheduled follow-up visit (6, 18, 30, 42, 54, and 66 months): it was assessed as the presence of ≥ Grade 2 clinical respiratory symptomatic patients and/or when it was observed on CT/X-ray performed for other reasons, according to Common Terminology Criteria for Adverse Events (CTCAE) score v.4.0, updated to v. 5.0 for all patients during the institutional reviews conducted in 2019 and subsequently in 2023.

Planning data (Eclipse Varian TPS v13.7) were retrieved, including 3D dose distributions, and exported into the MIM software system (MIM Inc, v7.2.8).

### Recovery of cardiac toxicity information and heart dosimetry

All cardiovascular events occurring during follow-up—including decline in left ventricular function, arrhythmias, vascular toxicities, conduction abnormalities, valvular heart disease, heart failure, ischemic heart disease, and cardiac-related deaths—were prospectively recorded by the treating radiation oncologist. Events were systematically assessed at scheduled follow-up visits at 6, 18, 30, 42, 54, and 66 months (32). Patients were subsequently followed for up to 10 years, with database updates performed in 2019 and again in 2023 to ensure completeness of long-term outcomes. The study was approved by the institutional ethics committee and registered on ClinicalTrials.gov (NCT03077191 and subsequent amendments).

For the main purpose of this work, cardiac events were classified into two categories: CAC_corr, the ones considered to be more directly correlated with the presence of calcifications (Coronary artery disease, Acute myocardial infarction, Congestive heart failure and heart valve disease); CAC_uncorr, the remaining ones (Conduction abnormality, Atrial premature beats, Atrial fibrillation, Pericarditis and Tachyarrhythmia). The distinction between the two categories was grounded in established pathophysiological principles and supported by cardiology literature. The CAC_corr group was defined on the basis of well-documented evidence that coronary artery calcification is a direct marker of atherosclerotic plaque burden and is strongly and independently predictive of these specific outcomes (37–44).

Conversely, the CAC_uncorr group represents conditions whose primary pathophysiology is not directly driven by atherosclerotic calcification, being mostly due to conduction disturbances and predominantly mediated by electrical remodelling, fibrosis, and inflammatory processes, and well-recognised as potential sequelae of thoracic radiation exposure (17, 18).

The heart was automatically segmented using a commercial AI-based tool (MIM Protégé), primarily to address the absence of heart contours in a proportion of right-sided cases and to minimize inter-observer variability. The mean heart dose (MHD) was considered the main dosimetric parameter of interest for cardiotoxicity and was therefore extracted for all patients. Moreover, the impact of MHD was also tested as dichotomic, taking the previously found optimal cut-off value (i.e.: 1Gy) (21).

### Extraction of quantitative CAC information

As a first step, patients with pacemakers, electrodes, or stents were identified through analysis of heart density histograms and automatic detection of high-density materials in the superior vena cava using TotalSegmentator (45). These patients (n = 81) were excluded, as their potentially altered baseline cardiac function could confound the interpretation of post-radiotherapy cardiac events. Cardiac calcifications (CAC) were subsequently identified using an in-house Python script (available at https://github.com/pymaitre/cluster_score.git) which classified calcified lesions as regions with attenuation values >130 HU and area ≥1 mm² or ≥4 contiguous pixels, in accordance with Neves et al. (28). The algorithm systematically screened the segmented heart for calcium deposits and included those located in the coronary arteries, cardiac valves, and myocardium. For each patient, the Agatston score (AS) (27), CAC_volume and HU score (Max_HU) were computed. Further details were reported elsewhere (21).

### Statistical analyses and modelling

After excluding patients with pacemakers, electrodes, or stents, a total of 1091 patients were available for model development. The association between the two categories of cardiac events and the available clinical and dosimetric variables was evaluated using logistic regression analysis. Initially, univariate logistic regression was performed for each variable. To assess inter-variable relationships and reduce redundancy, a Spearman correlation matrix was computed, with the aim of minimizing multicollinearity. In the presence of significant correlations between variables—excluding CAC-related metrics—only the variable with the lowest p-value was retained for further analysis. Subsequently, variables with a p-value < 0.20 in univariate analysis were considered for inclusion in multivariate logistic regression (MLR). Given the strong inter-correlation among calcification metrics, the three CAC scores were tested separately to generate models including only one CAC-related variable at a time. All analyses were conducted using an in-house Python repository routinely employed by our group (33). Model coefficients were estimated using maximum likelihood methods, applying a backward stepwise selection approach. Statistical significance was defined at the conventional threshold of p < 0.05. Model performance was assessed using receiver operating characteristic (ROC) curve analysis, with calculation of AUC, sensitivity, specificity, and accuracy. The final multivariable models were internally validated through bootstrapping, in accordance with TRIPOD type I recommendations (46)). Optimism-corrected AUC values were obtained from 1000 bootstrap iterations following the method proposed by Steyerberg (47).

## Results

Median follow-up was 6.5 years with IQR of 5.9-8.8 years. Overall, 29 patients experienced cardiac events, as previously reported in Belardo et al. (21) and in **Supplementary Table S1** of the **Supplementary Materials**.

As shown in **Supplementary Table S2** of the **Supplementary Materials**, 15 events were reported as CAC_corr events with a roughly balanced distribution between right and left breast cancer side, 8 vs 7 respectively (**Supplementary Table 3**). Regarding the remaining 14 events referring to the CAC_uncorr events, only one event was reported for right breast cancer patients (**Supplementary Table 4**). Due to this strong imbalance and the predominance of laterality (p-value = 0.0006, AUC = 0.71, **Supplementary Table S5** of the **Supplementary Materials**), CAC_uncorr analyses were restricted to the left breast patients only (13 events out of 561 patients).

Regarding respiratory toxicity, 18/1172 (1.5%) ≥G2 events were identified, all occurring within the first three years after radiotherapy and therefore well preceding the cardiac endpoint. The collected events included radiation pneumonitis, pleuritis, acute exacerbations of chronic obstructive pulmonary disease (COPD), pulmonary edema, and recurrent bronchitis, as reported in **Supplementary Table S6** of the **Supplementary Materials**.

**Tables 2** and **3** summarize the results of univariate analysis for both subgroups of patients. For CAC_corr, all three CAC scores were strongly significant predictors (p<0.001). Age was also significant while obesity and hypertension were of borderline significance.

**Table 2:** Summary of univariate analysis for events more related to cardiac calcifications (CAC_corr).

Variable	P-value	Odds ratio	CI at 95%
Laterality	0.7107	0.8247	[0.2970; 2.2897]
**Age**	**0.0014**	**1.0806**	**[1.0257; 1.1384]**
Chemotherapy	0.6259	0.7355	[0.2060, 2.6254]
Anthracyclines	0.2507	0.3563	[0.0464; 2.7372]
FEC	0.1424	0.0001	/
Concomitant CT/RT	0.3811	2.8838	[0.3655; 22.7523]
Hormonal therapy	0.1573	3.4094	[0.4462; 26.0482]
Lung Toxicities	0.2436	4.4491	[0.5532; 35.7797]
Smoking	0.5478	1.4396	[0.4540; 4.5648]
Obesity	0.1396	2.2607	[0.7970; 6.4121]
Diabetes	0.9623	0.9654	[0.2158; 4.3190]
Hypertension	0.3270	1.6780	[0.7970; 6.4122]
MHD	0.6771	0.9117	[0.5831; 1.4255]
1 Gy cutoff MHD	0.6674	1.2553	[0.4437, 3.5517]
**Agatson score**	**<0.0001**	**1.0017**	**[1.0011; 1.0023]**
**Volume score**	**<0.0001**	**1.0011**	**[1.0007; 1.0015]**
**MAX HU score**	**<0.0001**	**1.0040**	**[1.0022; 1.0059]**

Bold values indicate variables that are either statistically significant (p < 0.05) or included in the final multivariate model.

**Table 3:** Summary of univariate analysis for events less related to cardiac calcifications (CAC_uncorr), restricted to left breast cancer patients.

Variable	P-value	Odds ratio	CI at 95%
**Age**	**0.0591**	**1.0484**	**[0.9963; 1.1032]**
Chemotherapy	0.7815	0.8334	[0.2263, 3.0720]
Anthracyclines	0.6409	1.3775	[0.3722; 5.0982]
FEC	0.0800	3.8126	[1.0088; 14.4096]
Concomitant CT/RT	0.4323	0.0002	/
Hormonal therapy	0.3502	0.5524	[0.1670; 1.8273]
**Lung Toxicities**	**0.0005**	**27.1205**	**[5.9284; 124.0680]**
Smoking	0.2422	0.3468	[0.0044; 2.7005]
Obesity	0.9446	0.9550	[0.2589; 3.5218]
Diabetes	0.1235	2.7611	[0.8296; 9.1894]
Hypertension	0.3997	1.6151	[0.5352; 4.8740]
MHD	0.1062	0.6387	[0.3594; 1.1351]
1 Gy cutoff MHD	0.2236	/	/
**Agatson score**	**0.0120**	**1.0013**	**[1.0005; 1.0022]**
**Volume score**	**0.0157**	**1.0009**	**[1.0003; 1.0015]**
MAX HU score	0.0790	1.0023	[1.0000; 1.0046]

Bold values indicate variables that are either statistically significant (p < 0.05) or included in the final multivariate model.

MHD was not associated with cardiac events neither overall nor encoded using the best cut-off. The best multivariate model combined age and CAC scores with similar performances across the three scores (AUC = 0.80/0.79, p< 0.001), as shown in **Table 4**. The addition of respiratory toxicity in the analysis did not improve performance.

**Table 4:** Logistic regression model for Heart Toxicities prediction in group of events more related to cardiac calcifications (CAC_corr) when considering the three considered cardiac calcifications (CAC) scores one at time (from top to bottom: cardiac calcifications (CAC) volume, Agatson score (AS) and Max_HU).

Multivariate analysis for Heart_Toxicities in CAC_corr group						
ROC-AUC [Corrected]	Model p-value	Variables	Coefficient	p-value	OR	C.I. OR
0.81 [0.80]	p<0.0001	**Patient Age**	0.0475	0.0907	1.0486	[0.9925, 1.1079]
		**CAC Volume**	0.0009	<0.0001	1.0009	[1.0005, 1.0013]
		Constant	-7.8029	/	/	/
0.80 [0.79]	p<0.0001	**Patient Age**	0.0478	0.0897	1.0489	[0.9926, 1.1085]
		**AS**	0.0014	<0.0001	1.0014	[1.0008, 1.0021]
		Constant	-7.7852	/	/	/
0.79 [0.78]	<0.0001	**Patient Age**	0.0538	0.0447	1.0553	[1.0013, 1.1121]
		**Max_HU**	0.0033	0.0012	1.0033	[1.0013, 1.0053]
		Constant	-8.7425	/	/	/

Bold values indicate variables that are either statistically significant (p < 0.05) or included in the final multivariate model.

Regarding CAC_uncorr (restricted to only left breast patients), in the univariate analysis respiratory toxicity was a significant predictor, together with Age and CAC scores. MHD was of borderline significance. The best multivariate model considering only variables available before RT combined age and FEC (neo-adjuvant anthracycline-based chemotherapy) (AUC = 0.73, p=0.012).

The addition of respiratory toxicity in the analysis significantly improved performances (AUC = 0.77, p<0.001), as visible in **Table 5**.

**Table 5:** Logistic regression models for Heart Toxicities prediction in group of events less related to cardiac calcifications (CAC_uncorr) (left breast patients) when considering or not the Lung Toxicities variable.

Multivariate analysis for Heart_Toxicities in CAC_uncorr group (Left breast patients)						
ROC-AUC [Corrected]	Model p-value	Variables	Coefficient	P-value	OR	C.I. OR
0.73 [0.71]	0.0125	**Patient Age**	0.0652	0.0252	1.0673	[1.0081, 1.1300]
		**FEC**	1.9300	0.0103	6.8892	[1.5765, 30.1058]
		Constant	-8.1755	/	/	/
0.77 [0.73]	0.0003	**Patient Age**	0.0552	0.0590	1.0568	[0.9979, 1.1191]
		**FEC**	1.8177	0.0214	6.1580	[1.3092, 28.9651]
		**Lung Toxicity**	3.1165	0.0002	22.567	[4.3705, 116.5210]
		Constant	-7.7383	/	/	/

Bold values indicate variables that are either statistically significant (p < 0.05) or included in the final multivariate model.

**Figure 1:**
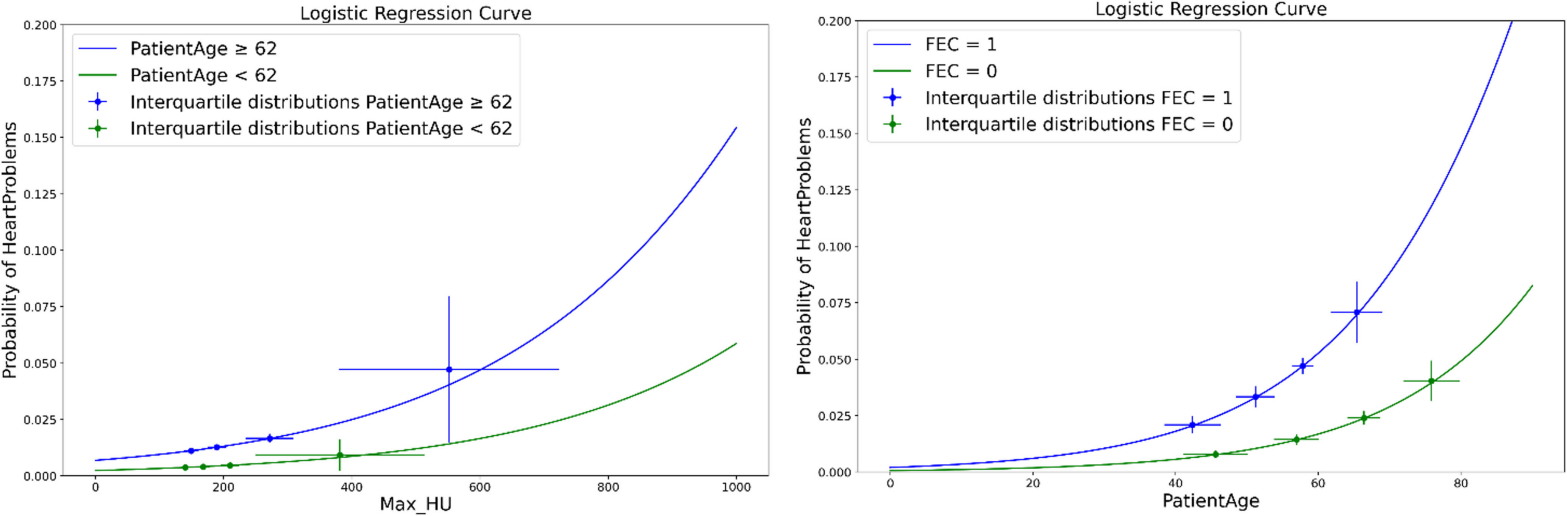
On the left, logistic regression for group of events less related to cardiac calcifications (CAC_corr) with curves splitting for Patient Age median of 62y. The model considered both right and left breast cancer patients. On the right, logistic regression for group of events less related to cardiac calcifications (CAC_uncorr) with curves splitting for patients who undergoing FEC (Fluorouracil, Epirubicin, and Cyclophosphamide) or not. The model considered only Left breast cancer patients.

**Figure 1** shows on the left the predicted risk as a function of Max_HU stratified against Patient Age, above and below the median value of 62 years. On the right the predicted risk as a function of Patient Age splitting for usage of FEC or not is showed.

Internal validation confirmed the robustness of the models, with AUC correction for the optimism within 0.03. For example, the model using Max_HU showed AUC = 0.78 compared to the original value of 0.79.

## Discussion

Starting from our previous work (21) in which different cardiotoxic events were considered as a single endpoint, the question naturally arose as to whether specific predictors could be associated with different subgroups of cardiac events.

Increasing evidence suggests that CAC represents major predictors of cardiac toxicity after breast cancer radiotherapy and are also associated with reduced overall survival (22–26, 29).

Regarding the predictive value of CAC scores on selected types of CVD events, Huangfu et al. (37) analyzed the Coronary artery calcification - dispersion and density (CAC-DAD) in relationship with only major adverse cardiovascular events (MACE), defined as nonfatal myocardial infarction or cardiovascular mortality. They reported that an elevated CAC-DAD score was significantly associated with MACE (P<0.001) on Kaplan-Meier analysis. On univariable testing, an elevated CAC-DAD score (HR, 3.30 [95% CI, 1.86–5.86]; P<0.001) was significantly associated with MACE. Age, diabetes, hypertension, and statin use were also significant predictors. The area under receiver operating characteristic of the resulting multivariable model containing the CAC-DAD score was moderately high (AUC = 0.65; P = 0.003).

Our investigation builds on previous efforts aimed at clarifying the complex interplay among breast cancer treatments, CAC and cardiac dosimetry, with particular attention to the identification of predictors of cardiac toxicity that are expected not to be primarily driven by CAC burden. While CAC emerged as strong markers of, mostly sub-clinical, baseline cardiovascular conditions, the determinants of cardiac events occurring independently of CAC severity remain less clearly defined and underexplored. In this context, our analysis sought to identify treatment-related and dosimetric predictors that may contribute to cardiotoxicity beyond the effect of pre-existing calcifications.

We deliberately focused on a cohort of patients treated with moderate hypofractionation (40 Gy in 15 fractions) to extend current knowledge to this nowadays standard treatment regimen, while also reflecting the most common clinical scenario of whole-breast irradiation alone.

First, based on previously cited studies (37–44) that confirm a common mechanistic basis rooted in atherosclerotic plaque burden and vascular calcification, for which CAC severity is a well-validated surrogate and independent predictor, we grouped together events considered CAC-related (CAC_corr). Within this subgroup, which accounted for 15 of the 29 observed cardiac events, the main predictors were CAC scores and age, yielding strong discriminative performance (AUC = 0.80/0.79), while laterality and cardiac dosimetry did not show any significant impact.

CAC-related metrics emerged as leading predictors (p<0.001). The inclusion of respiratory toxicity variables did not improve the performance of the two-variable model based on age and CAC burden, as respiratory toxicity was not significantly associated with the outcome in univariate analysis (p = 0.2436).

Second, the remaining events were classified as CAC-unrelated (CAC_uncorr), based on the well-established evidence that their primary pathophysiology involves electrical remodeling, myocardial fibrosis, or radiation-induced inflammatory processes rather than direct atherosclerotic mechanisms (17, 18). Within this group, which accounted for 14 of the 29 events, a striking effect of laterality (and of MHD>1Gy) immediately emerged: 13 of the 14 events occurred in patients with left-sided breast cancer (561 out of 1091 patients). In order to more accurately investigate the actual predictors underlying this subgroup of events, a more restricted analysis was focused to patients with left-sided tumors only. CAC_uncorr events seemed to depend mostly on age and anthracyclines. This result is consistent with others, as, for instance, recently summarized by Lyon et al. (17, 18), Cancer therapy-related cardiac dysfunction during anthracycline chemotherapy may present clinically or be detected in asymptomatic patients during surveillance. The diagnosis of anthracycline chemotherapy-related cardiac dysfunction includes new Cardiovascular (CV) symptoms, new abnormalities in cardiac function on CV imaging, and/or new increases in cardiac biomarkers.

Once restricted the analysis to left breast cancer patient, MHD was not an impacted predictor, in line with other modern series where heart sparing is much more efficient than in the past, substantially compressing the inter-patient variability in MHD and thereby reducing the statistical sensitivity to detect any dose-response relationship (13, 14, 48, 49). This finding is consistent with our previous work on the same cohort (21), in which MHD as a continuous variable was not independently confirmed either, and was only informative when dichotomized at a cut-off of 1 Gy — a threshold that largely reflects laterality. The absence of a significant MHD association in this setting should therefore not be interpreted as evidence against a radiation dose effect in general, but rather as a consequence of the low MHD and of its limited inter-patient variability achieved by modern heart-sparing radiotherapy techniques in whole-breast-only irradiation.

CAC_uncorr analyses also suggest an interplay with moderate/severe respiratory problems occurring in the first 3 years after Radiotherapy likely mediated by a mechanism like that observed after respiratory infections. This result was reported, to our knowledge, for the first time in a breast cancer cohort: model’s performance significantly improved from AUC = 0.73, p=0.012 up to AUC = 0.77, p<0.001.

Overall, direct comparison across the limited number of studies investigating the relationship between CAC and cardiac radiation dose remains difficult. This is largely attributable to heterogeneity in endpoint definitions and, importantly, to differences in the methods used for collecting cardiac events. In this context, a limitation of our study lies in the combined prospective and retrospective collection of clinical data, which may have resulted in under-detection of events. As widely acknowledged, capturing cardiac outcomes is inherently challenging due to the long follow-up required; moreover, even registry-based data are not exempt from under-reporting (25). Consequently, both the total number of observed events and their timing may be affected by a certain degree of uncertainty.

An additional point to consider when interpreting our findings is the relatively small number of events available for analysis. This constraint necessitated subgroup analyses which, although informative for hypothesis exploration, may introduce statistical instability and limit the strength of the conclusions. Therefore, the present results should be interpreted with caution. Although penalized regression approaches (e.g., Firth correction, LASSO) were not applied in the present analysis, evidence from our group suggests that, in comparable settings, logistic regression models with a parsimonious feature set achieve performances similar to more complex penalized and machine learning approaches (33).

Despite these limitations and the methodological variability across studies, findings from different groups have shown a reassuring degree of consistency. An ongoing multicentric prospective trial (50) is expected to provide more robust evidence and may support the integration of CAC assessment from planning CT scans into routine clinical practice.

Another limitation of the current study is the lack of a detailed analysis of dose distribution to specific cardiac substructures, as well as the absence of information on the spatial localization of CAC. Within an EU-funded project (51) we planned to expand these analyses by pooling our dataset with three additional large cohorts, reaching a total of more than 4000 patients. This effort is expected to provide further insight into the interaction between CAC and spatial dose patterns. However, it should be acknowledged that uncertainties in the delineation of cardiac substructures—particularly the left anterior descending artery (LAD)—remain a critical issue and may contribute to the variability of results reported in the literature.

The importance of evaluating baseline cardiovascular risk is increasingly emphasized, as highlighted in the recent ESC cardio-oncology guidelines (19). Advancing the understanding of dose–volume relationships and developing predictive models that integrate both treatment-related and patient-related factors represent key priorities in cardio-oncology research. Although modern radiotherapy techniques have reduced cardiac exposure, placing most patients in a relatively low-risk category, the high global incidence of breast cancer makes this issue clinically significant. Beyond improving treatment planning and delivery, early identification of patients at higher risk is essential to enable personalized follow-up strategies.

## Conclusions

Our results suggest for the first time that coronary artery calcification (CAC)-related and CAC-unrelated cardiac events may be driven by partially different mechanisms translating into different major predictors. CAC_corr events appear to be predominantly influenced by baseline cardiovascular risk factors, captured by CAC burden and age as potential surrogate of pre-existing cardiovascular vulnerability. Conversely, CAC_uncorr events showed a markedly different pattern. Almost all events were registered for left breast cancer patients, identifying in this way a family of events that are mostly due to Radiotherapy. Once restricting the analysis to left breast patients, age and exposure to anthracyclines appeared to play the most relevant roles, highlighting the multifactorial nature of treatment-related cardiac risk.

Interestingly, our results also suggest a possible association between CAC_uncorr events and the previous occurrence of moderate/severe respiratory toxicity within the first three years after radiotherapy. However, this observation should be interpreted with caution due to the limited number of events and the exploratory nature of the analysis.

## Data Availability

The raw data supporting the conclusions of this article will be made available by the authors, without undue reservation.
