# Supplementary material for: Evidence of different predictors for different types of cardiotoxicities after hypo-fractionated whole breast radiotherapy
Source: Front Oncol. 2026 Jul 22;16:1893940. doi: 10.3389/fonc.2026.1893940 (PMC13437494; doi:10.3389/fonc.2026.1893940)
Supplement: Supplementary file 1 [file Table1.docx]

**Supplementary materials**

**Table 1:** Detailed list of cardiac events per patient.

**Heart problems’ description**

Coronary artery disease (CAD)

Conduction abnormality

Atrial premature beats (APB)

Atrial fibrillation (AF)

Acute myocardial infarction (AMI)

Pericarditis

Congestive heart failure (CHF)

Tachyarrythmia

Heart valve disease (HVD)

|  | **Problem 1** | **Problem 2** | **Laterality** | **Age (Years)** | **Months from end of therapy** | **Lung Toxicity** |
| --- | --- | --- | --- | --- | --- | --- |
| **Patient 1** | AF | - | Left | 71 | 24.4 | No |
| **Patient 2** | Pericarditis | - | Left | 66 | 5.1 | Yes |
| **Patient 3** | CHF | - | Right | 62 | 66.0 | No |
| **Patient 4** | AF | - | Left | 69 | 73.0 | No |
| **Patient 5** | Tachyarrythmia | - | Left | 74 | 63.9 | No |
| **Patient 6** | Pericarditis | - | Left | 46 | 12.2 | No |
| **Patient 7** | APB | - | Left | 70 | 48.7 | No |
| **Patient 8** | AMI | - | Left | 72 | 29.4 | No |
| **Patient 9** | HVD | - | Left | 58 | 40.5 | No |
| **Patient 10** | AF | - | Left | 58 | 80.2 | No |
| **Patient 11** | AMI | - | Right | 69 | 83.2 | No |
| **Patient 12** | AMI | - | Right | 75 | 51.7 | No |
| **Patient 13** | CHF | HVD | Left | 66 | N/A | No |
| **Patient 14** | CHF | AMI | Left | 80 | 101.4 | No |
| **Patient 15** | AF | - | Left | 72 | 19.6 | No |
| **Patient 16** | CHF | - | Left | 79 | 60.5 | No |
| **Patient 17** | CHF | - | Right | 46 | 98.4 | No |
| **Patient 18** | AF | - | Left | 74 | 96.3 | No |
| **Patient 19** | CAD | - | Right | 70 | 86.2 | No |
| **Patient 20** | AMI | - | Right | 70 | 53.4 | No |
| **Patient 21** | CAD | - | Right | 67 | 62.9 | No |
| **Patient 22** | Conduction abnormality | - | Left | 80 | 78.2 | No |
| **Patient 23** | Tachyarrythmia | - | Left | 54 | N/A | No |
| **Patient 24** | AMI | - | Left | 69 | 91.4 | No |
| **Patient 25** | AF | - | Left | 64 | 31.4 | No |
| **Patient 26** | Conduction abnormality | - | Left | 78 | 93.5 | Yes |
| **Patient 27** | AF | CHF | Left | 69 | 36.5 | Yes |
| **Patient 28** | AF | CHF | Right | 77 | 49.7 | No |
| **Patient 29** | AF | CHF | Right | 70 | 82.2 | Yes |

**Table 2:** Summary of grouped cardiac events. In yellow the events considered more directly related to the presence of cardiac calcifications (CAC) (CAC_corr, n=18 of 15 patients). The remaining are the ones not considered to be directly related to CAC (CAC_uncorr, n=16 of 14 patients).

| **Heart problem** | **N^o^ of events** |
| --- | --- |
| Congestive heart failure (CHF) | 8 |
| Acute myocardial infarction (AMI) | 6 |
| Coronary artery disease (CAD) | 2 |
| Heart valve disease (HVD) | 2 |
| Atrial fibrillation (AF) | 9 |
| Conduction abnormality | 2 |
| Pericarditis | 2 |
| Tachyarrythmia | 2 |
| Atrial premature beats (APB) | 1 |

**Table 3:** Summary of the major clinical characteristics of the patients whose heart problems were more directly related to the presence of cardiac calcifications (CAC).

| **Total number of patients** | 15 |
| --- | --- |
| **Age Groups** |  |
| Mean  Median [IQR]  < 45 | 70  71[69;77]  0 |
| 45 - 55 | 1 |
| 55 - 65 | 1 |
| > 65 | 13 |
| **Position of tumour** |  |
| Left | 7 |
| Right | 8 |
| **Chemotherapy** |  |
| Anthracycline based | 1 |
| Other | 2 |
| Concurrent Chemo/RT | 1 |
| **Other therapies** |  |
| Trastuzumab | 0 |
| Hormonal | 14 |
| **Health conditions** |  |
| Obesity | 6 |
| Diabetes | 2 |
| Hypertension | 7 |
| Smoking | 4 |
| **Mean Heart Dose (MHD)** |  |
| Average (SD) [Gy] | 1.59 (1.05) |
| Patients with MHD > 1 Gy | 9 |

**Table 4:** Summary of the major clinical characteristics of the patients whose heart problems were more directly related to the laterality and Mean Heart Dose (MHD).

| **Total number of patients** | 14 |
| --- | --- |
| **Age Groups** |  |
| Mean  Median [IQR]  < 45 | 66  69[62;72]  0 |
| 45 - 55 | 2 |
| 55 - 65 | 3 |
| > 65 | 9 |
| **Position of tumour** |  |
| Left | 13 |
| Right | 1 |
| **Chemotherapy** |  |
| Anthracycline based | 3 |
| Other | 1 |
| Concurrent Chemo/RT | 0 |
| **Other therapies** |  |
| Trastuzumab | 2 |
| Hormonal | 9 |
| **Health conditions** |  |
| Obesity | 4 |
| Diabetes | 4 |
| Hypertension | 6 |
| Smoking | 2 |
| **Mean Heart Dose (MHD)** |  |
| Average (SD) [Gy] | 1.97 (0.95) |
| Patients with MHD > 1 Gy | 13 |

**Table 5:** Summary of univariate analysis for events less related to cardiac calcifications (CAC_uncorr).

| **Variable** | **p-value** | **Odds ratio** | **CI at 95%** |
| --- | --- | --- | --- |
| **Laterality** | **0.0006** | **12.5539** | **[1.6363; 96.3147]** |
| Age | 0.0672 | 1.0442 | [0.9953; 1.0955] |
| Chemotherapy | 0.7796 | 1.1839 | [0.3683, 3.8055] |
| Anthracyclines | 0.6292 | 1.3868 | [0.3830; 5.0215] |
| FEC | 0.0762 | 3.8056 | [1.0383; 13.9489] |
| Concomitant  CT/RT | 0.4070 | 0.0259 | / |
| Hormonal therapy | 0.1518 | 0.4283 | [0.1421; 1.2914] |
| **Lung Toxicities** | **0.0010** | **19.3458** | **[4.8950; 76.4580]** |
| Smoking | 0.5598 | 0.6561 | [0.1461; 2.9461] |
| Obesity | 0.6273 | 1.3442 | [0.4179; 4.3232] |
| Diabetes | 0.1459 | 2.5513 | [0.7897; 8.2422] |
| Hypertension | 0.5127 | 1.4332 | [0.4936; 4.1615] |
| MHD | 0.4552 | 1.1636 | [0.7937; 1.7059] |
| **1 Gy cutoff MHD** | **0.0010** | **11.6584** | **[1.5193; 89.4598]** |
| **Agatson score** | **0.0297** | **1.0010** | **[1.0003; 1.0018]** |
| **Volume score** | **0.0439** | **1.0006** | **[1.0001; 1.0011]** |
| MAX HU score | 0.0666 | 1.0025 | [1.0001; 1.0048] |

**Table 6:** Summarised pulmonary events post-radiotherapy

| **Pulmonary event** | **N^o^ of events** |
| --- | --- |
| Radiation pneumonitis | 4 |
| Non-radiation pneumonia, inflammation/infection episodes | 4 |
| Bronchitis / bronchiectasis | 3 |
| Pulmonary fibrosis | 2 |
| COPD / emphysema / asthma-related | 1 |
| Atelectasis | 1 |
| Pleuritis accompained to pericarditis | 1 |
| Pulmonary embolism | 1 |
| Pulmonary edema | 1 |
